# Nanostructured Gels for Energy and Environmental Applications

**DOI:** 10.3390/molecules25235620

**Published:** 2020-11-29

**Authors:** Maria Cristina Cringoli, Silvia Marchesan, Michele Melchionna, Paolo Fornasiero

**Affiliations:** 1 Chemical & Pharmaceutical Sciences Department, University of Trieste, 34127 Trieste, Italy; mcringoli@units.it (M.C.C.); smarchesan@units.it (S.M.); 2 INSTM Trieste Research Unit, 34127 Trieste, Italy; 3 ICCOM-CNR Trieste Research Unit, 34127 Trieste, Italy

**Keywords:** gels, nanomaterials, energy, water sustainability, hydrogen production, supramolecular gels, polymeric gels

## Abstract

Nanostructured gels have emerged as an attractive functional material to innovate the field of energy, with applications ranging from extraction and purification to nanocatalysts with unprecedented performance. In this review we discuss the various classes of nanostructured gels and the most recent advancements in the field with a perspective on future directions of this challenging area.

## 1. Introduction

In an ever-growing technological society, design and development of new materials for use in many fields, including automotive engineering for the design of all-electric vehicles, military, aviation and aerospace industries, need to address many demands and requirements for ensuring competitiveness and industrial transfer. For example, new functional materials should bear high capacity and enhanced performances in energy storage and conversion systems, such as supercapacitors and batteries, so as to facilitate implementation of new, sustainable and green production processes.

**Figure 1:**
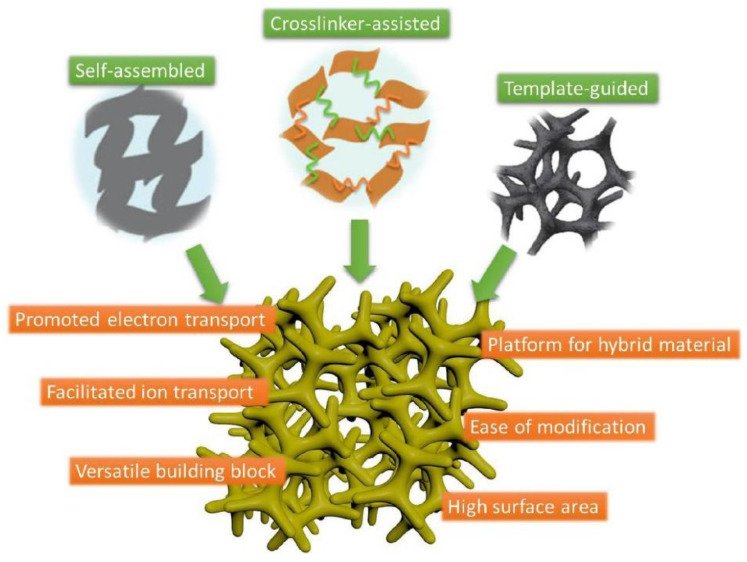
Advantageous features of gel-based nanomaterials for energy-related applications. Reprinted from ref. [3], Copyright 2016, with permission from Elsevier [3].

Traditional battery systems are typically based on active materials and conductive additives randomly mixed in a non-conductive polymer binder that ensures the interactions between all the electrode units and the metal component. In this way, electrons can deftly move through the composite material towards the metal current collector. The weakness of such a system is usually related to poor mechanical properties and flexibility of the binders that hinder the effective contacts among all the regions. As a result, life cycle and efficiency of the battery are decreased [1,2]. For these reasons, optimisation of binders is essential to obtain new generations of electrodes with highly improved performances, and characterised by good mechanical strength and elastic properties, low resistance and high electronic conductivity. Several approaches have been employed to address these issues, such as the design of conductive polymers, playing the double role of conductive additives and adhesive binders. In this context, gel systems are an emerging class of materials that can provide longer stability and enhanced electrochemical properties, owing to their unique three-dimensional (3D) network, which can be fine-tuned up to nanoscale level [2]. Gels offer the possibility to act as soft binders between the different battery components, and their viscoelastic properties can be valuably exerted to coat flexible and wearable electronic devices. On the other hand, gels can also support the advantages of nanotechnology for higher reactivity, by both incorporating nanocomponents and acting as nanosized matrices (Figure 1) [3].

This review aims at providing the most recent developments in energy field, whereby gels are used as innovative constituents of nanomaterials for energy applications. The discussion starts with a general description of the properties and the improvements conferred by gelling materials to electronic devices. Then, it is organised by the different applications, from water management with a specific focus on the fabrication of multifunctional metal nanomaterials, to the field of nano-electrocatalysis for energy generation and harvesting.

## 2. Gels: Definitions and Diversity

Gels are soft materials made of at least two components, one of which is liquid and the most abundant (at least 95% of the entire material), while the other one is the gelator. Based on the nature of the solvent, they can be named hydrogels, if the liquid phase is constituted by water, or organogels, whereby organic solvents are used. Gelators molecules can form 3D networks established on either non-covalent interactions (physical gels) or chemical bonds (chemical gels). In the context of physical gels, they can be based on weak interactions (e.g., hydrogen bonding, hydrophobic interactions, salt-bridge interactions) and self-assembling processes to form the so-called supramolecular gels. On the other hand, chemical covalent bonds result in stronger gels, involving cross-linking reactions, condensation and grafting processes [4,5]. Consequently, different classes of gelators can be employed to provide diversity in gel attributes: polymers, carbon nanomaterials, metals, inorganic compounds, small organic molecules are some examples of the wide range of chemical species that can offer the possibility to create networks with highly defined mechanical properties and chemical responsiveness [3].

With regard to energy devices for conversion and storage applications, gels can impart several valuable improvements. In particular, the well-defined gel network can act as an advantageous platform for both electron transport and ion diffusion, which are two critical processes for proper material functionality. The second benefit brought by the 3D network builds on its soft nature that ensures accommodation of volume changes, typically occurring during electrochemical processes. In this way, electrode breaks are prevented and the correct interactions among all the components are assured [2]. The softness of a gel derives from its viscoelastic properties, which can be measured in elastic and viscous components, namely G’ and G” modulus, respectively. These parameters can be tuned by changing different factors, including gelator type and concentrations, pH, temperature, gelling methods. Accurate modifications can be assessed to create flexible materials, useful for coating innovative and stretchable electronic devices [3,6,7]. Furthermore, nanoparticles can be dispersed in gel phase. Indeed, nanotechnology here is crucial as, over the last two decades, it has importantly contributed to enhance performances of energy storage and conversion devices, increasing many properties such as reaction surface area, capacity and stability [8]. Gels can improve nanoparticle potentials in different ways. For example, they can be used to conveniently stabilise nanoparticle aggregation and sedimentation. For example, it has been reported that agarose-based gels are valuable green candidates to act as inert matrices to prevent cerium oxide (ceria, CeO_2_) nanoparticle sedimentation processes in aqueous media [9]. In fact, CeO_2_ nanoparticles are largely investigated for fuels cells and photoelectron applications, where they are commonly present as catalysts or supports to increase cell performances, in combination with other metals, carbon nanoparticles or polymers [10,11]. To the best of our knowledge, only few applications of ceria-based nanoparticles embedded in wet gel matrix have been reported [12,13], but we envisage that gels could be used to further extend the potentials of such powerful nanomaterials, particularly for electrocatalytic applications.

The use of gels in energy-related fields could also refer to an issue particularly hot for the current society: the interconnection among energy sources, sustainability and pollution. Indeed, many efforts have been made to investigate new greener methodologies and bypass the heavy energy dependence from fossil fuels [8]. Hydrogels, which are typically characterised by high biocompatibility being mainly made of water, represent a precious opportunity for the advancement of clean energy schemes, favouring green and sustainable materials over processes with high environmental impacts.

### 2.1. Supramolecular Gels: Self-Assembly and Triggers

Non-covalent interactions between small molecules can lead to nanofibrillar networks that sustain the formation of supramolecular gels. Typical gelators used are amphiphilic molecules that can be designed on aliphatic (as in the case of long-chain hydrocarbons, surfactants, amides, ureas, peptide derivatives) or on aromatic backbones (such as pyrene, perylene, porphyrins derivatives). In the latter case, aromatic moieties offer more favourable interactions, based on π–π stacking that add up to H-bonding, van der Waals, dipole–dipole and electrostatic forces. As a result of the self-assembling process, nanostructured networks based on nanofibers, nanotubes, nanosheets, or nanospheres are achieved [14,15]. In this case, thus, the gel matrix is confined to the nanoscale, offering a high internal surface area, improving reactivity and the number of available pathways for electron and ion transport [3]. The most popular strategies that can be applied to obtain supramolecular gels include thermally induced gelation (by heating a saturated solution that is converted to gel upon cooling to room temperature), sonication, solvent switches, or pH triggers for hydrogels. [14]. The use of light irradiation to induce self-assembly is remarkable for spatially resolved gels [16]. In this case the localised gelation can be obtained by the use of gelators bearing photo-switchable moieties, including azobenzene, stilbene, or spiropyran derivatives. Photomasks, which partially cover the materials from light irradiation with a specific pattern, allow the identification of selected and shaped areas that are designed to gel [15,17,18,19,20].

An innovative approach to control the self-assembly of a gel is the surface-mediated patterning [21,22,23]. In this case, an electrochemical trigger is used to induce a pH-shift in a solution of the gelator, which is converted into a hydrogel starting from the surface of the electrode. Different gel thicknesses can be achieved by merely changing the intensity and the time of the applied current. A high spatial resolution is thus realised, allowing nanostructured hydrogels with different regions of patterning and functionality [21]. This method has been recently employed to create nanoelectrode arrays for biosensing applications. Indeed, a nanostructured hydrogel electrochemically grown on the surface of microsquare nanoband edge electrode (MNEE) arrays has been reported to preserve the electrode-sensing ability to small molecules such as nucleic acids, and confer it, moreover, antifouling properties, useful for decreasing noise and interferences in favour of higher sensitivity [24].

### 2.2. Polymeric Gels and Their Tuneble Properties

Different monomers can be polymerised and then cross-linked to form networks that entrap large amount of solvent, which results in gels. Cross-linking strategies can be applied to small molecules, biomacromolecules, metals, or nanoparticles to yield different gels with different viscoelastic properties. Electrostatic interactions, metal coordination, or various chemical reactions are involved, including, among others, radical polymerisation, condensation reactions and click chemistry [25]. With respect to supramolecular gels, polymeric ones are typically characterised by higher mechanically stability and easy tuneable strength for the creation of gel materials with specific properties [26].

For instance, a captivating feature for cutting-edge electronic devices is the adaptability to deformations for practical flexible applications, maintaining, however, the electrical conductivity under the applications of the deformation [27]. Design of stretchable devices are generally based on conveniently cross-linked elastic natural polymers (e.g., elastin, alginate, chitosan) or on hybrid/composite gels containing nanomaterials (including inorganic nanoparticles or carbon-based nanomaterials) [28,29,30,31,32,33]. The encapsulation of metal nanocomponents, such as silver nanowires, into a polymer gel is a valuable strategy to obtain electronic conductors, with applications in innovative devices like human motion energy harvesters [32]. Other reported examples showed hydrogels based on long-chain polymers that have been chemically cross-linked onto silanised titanium wire surfaces. The obtained material was transparent and highly stretchable, allowing multiple cycles of stretches without modifying the electric resistance. Such a methodology paved the way to incorporate more complicated systems, such as arrays of light emitting diode (LED), into hydrogel matrix to yield soft and flexible electronic devices [34].

These remarkable properties are also exploited in energy storage devices, such as supercapacitors [35]. Polyaniline (PANI) hydrogels could be valuable conductive candidates for such applications [36], but they typically lack good cycle stability, prompting the synthesis of PANI composite hydrogels bearing carbon nanomaterials [37]. Indeed, the combination with graphene oxide (GO) has been extremely attractive to confer both improved electrochemical conductivity and flexibility to PANI gels [38,39]. The resulting nanostructured materials showed not only high electrochemical performances with good capacitive behaviour, but also an excellent stability to flexing cycles: 2000 mechanical bending cycles did not affect the capacitance of the composite gels [40].

**Figure 2:**
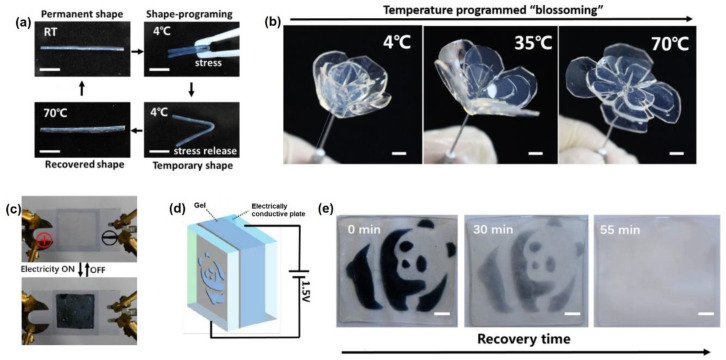
(**a**) Shape programming cycle of the composite hydrogel: an initial rodlike sample is deformed at room temperature by external stress and fixed in the shape upon cooling at 4 °C. When heated to 70 °C, the original rodlike shape is recovered. (**b**) Artificial three-dimensional hydrogel ‘flower’ is ‘blooming’ triggered by temperature changes. Photograph (**c**) and schematics (**d**) of the composite hydrogel placed between two electrically conductive plates used for the creation of (**e**) reversible printed pattern upon voltage application. Scale bars: 5 mm. Adapted with permission from ref. [43]. Copyright 2018 American Chemical Society [43].

Many interesting features can be conferred by polymer-based gels to electronic devices, including ability to respond to different stimuli, such as temperature, light, electricity and self-healing behaviour upon the application of external forces and damages [41,42]. Combination of many of these properties can create ‘smart’ functional gel materials that can undergo changes in their physiochemical properties in a dynamic manner. As an example, it has been recently reported that a composite hydrogel based on natural and synthetic biopolymers (chitosan, agarose and polyacrylamide) and polyoxometalate (SiW, silicotungstic acid) nanoparticles not only showed high stretchable and twistable abilities, but it also displayed a broad range temperature responsiveness, whereby temperature increases were related to decreases of gel stiffness. This particular thermal behaviour was thus exploited for shape programming and recovery to create shape memory materials (Figure 2a). The process was highly reproducible and resistant to many cycles, allowing hydrogel to be moulded in different three-dimensional objects, such as a ‘flower’ able to thermally trigger ‘blooming’ (Figure 2b). Furthermore, the presence of SiW nanoparticles conferred a redox activity to the gel, which was also electrochemically responsive. Indeed, when the composite gel was positioned between two conductive plates and a voltage was applied, a reversible change in the colour of the hydrogels was observed (as showed in Figure 2c–e). In this way, dynamic hydrogels with reversible and programmable behaviours have been obtained with significant possible applications in soft robotics and electricity sensors [43].

## 3. Applications

### 3.1. Water Purification and Metal Rebirth

Water shortage and lack of access to clean water are issues that affect at least 1.1 billion people worldwide [44]. Causes are imputable, among others, to human population growth, poverty, pollution and climate changes [45,46]. Management of waste and pollution control (including microbiological hazards) in water should consider the way matter and energy cyclically move in the environment and maintain their equilibrium through the atmosphere and the hydrosphere. In the field of energy conversion and storage, waste of electrical and electronic equipment (WEEE) are made of different hazardous components, in particular pollutant metals (e.g., palladium, nickel, gold, lithium) that can affect freshwater access. Moreover, the rise in temperatures due to global warming can increase aqueous solubility of metal ions and their concentrations in waters, causing not only higher exposure to toxic conditions from more organisms, but also their conversion in different chemical species that alter the biogeochemical cycle [47]. Beside sustainable and responsible water management and conservation, scientific developments are designing efficient methodologies for water treatments that are useful not only to sequestrate metal ions, but also to give them a second life as new gelling nanomaterials for energy applications.

**Figure 3:**
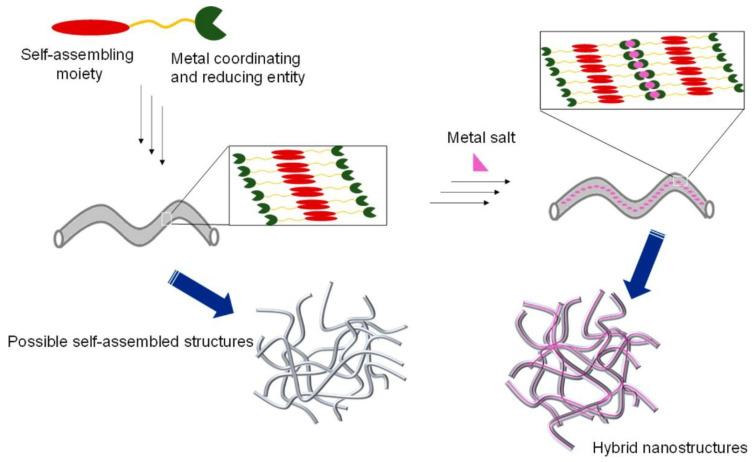
General scheme of design strategy for in situ synthesis of metal nanoparticles embedded in nanogel. Reprinted with permission from ref. [50]. Copyright 2016 American Chemical Society [50].

For instance, nanostructured hydrogels have been recently reported to scavenge palladium ions and induce its reduction to form Pd nanoparticles (PdNPs), without the aid of any reducing agent. Specifically, the gelator based on a sorbitol derivative self-assembled in the presence of agarose to give a hybrid hydrogel that was able to effectively uptake palladium ions from the surrounding solution. The scavenging activity was highly efficient, as the final Pd concentration in the solution was well below the legal limits imposed by health agencies to avoid metal toxicity. Moreover, authors observed the formation of spherical PdNPs with an average diameter of 5 nm, close to the hydrogel nanofibers, whereby an in situ reduction of Pd^2+^ to Pd^0^ occurred spontaneously at room temperature. The innovation of such a result lays in the fact that this method for NPs synthesis did not require the addition of external reducing agents (for example, citric acid) that are typically mandatory for the formation of metal nanoparticles. The effective production of PdNPs was also successfully tested by using the nano-hydrogel containing NPs as catalyst in Suzuki–Miyaura reactions, with excellent recyclability properties [48]. The same approach was also applied to separate other precious metal ions, i.e., Ag^+^ and Au^3+^, from aqueous environments, yielding the corresponding nanoparticles embedded in the hydrogel phase (see Figure 3) [49]. Moreover, this method showed a good selectivity for precious metals (Ag, Au, Pd and Pt) over other more abundant metals (such as Cu, Fe, Ni and Zn), as a result of the higher reduction potentials of the former [50,51].

**Figure 4:**
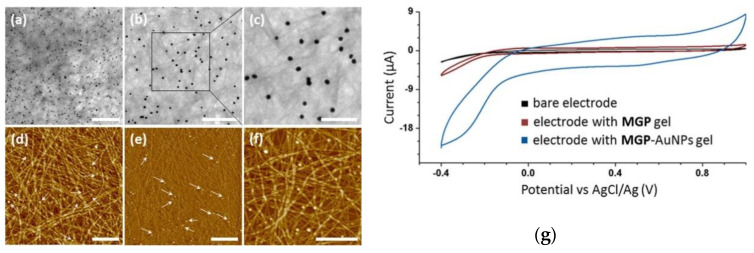
TEM (**a**–**c**) and AFM (**d**–**f**) images of AuNPs hybrid nanogel and cyclic voltammetry (**g**) of bare electrode (black) and electrode modified with nanostructured hydrogel in the absence (red) and in presence (blue) of AuNPs. Scale bars are 1 μm for (**a**,**d**,**e**), 500 nm for (**b**,**f**), and 200 nm for (**c**). Adapted with permission from ref. [52]. Copyright 2018 American Chemical Society [52].

Furthermore, the ordered organisation of metal NPs immobilised on the matrix nanofibers (see Figure 4a) provided intrinsic conductivity properties to the hydrogels, which can be used to modify the electrodes and favour electrocatalysis [51,52]. Indeed, cyclic voltammetry experiments were carried out to assess the O_2_ reduction catalysed by hybrid nanogels containing gold nanoparticles (AuNPs) obtained with this methodology. A carbon paste electrode (CPE) was thus modified with AuNPs hybrid gel and its reductive current was compared to those generated by the bare CPE and CPE modified with gel in absence of AuNPS. As reported in Figure 4g, the CPE modified with AuNPs nanogel showed a large reductive current, confirming the ability of this material to electrically wiring the carbon electrode [52]. The potentials of nanostructured hydrogels are hence demonstrated, as they can be useful candidates not only for the green uptake of toxic metals derived from electronic waste (such as palladium ions), but also for being employed as valuable templates for NPs synthesis and their subsequent use as heterogeneous organo- and electro-catalysts [48,51,52,53,54].

If the purification of waters from metal ions with these emerging technologies is undoubtably of relevant importance, on the other hand, the extraction of metal nanoparticles from aqueous waste is equally crucial to protect environment and living systems. Indeed, nanoparticles could induce severe toxicity as well, in particular if they are produced on large scale. For example, titanium dioxide (titania) nanoparticles (TiO_2_NPs) are among the most produced nanomaterials. This is not surprising as TiO_2_NPs find large applications in different fields, spanning from skincare products, pharmaceutical additives, anticorrosive agents, to photocatalysis. Titania NPs are benchmark semiconductors, extensively investigated in the photocatalytic hydrogen production from water-splitting processes. The presence of four different polymorphs (anatase, brookite, rutile and titania B) allows various applications, as each structure is characterised by peculiar electronic properties and, thus, different photocatalytic features [55,56,57,58]. Moreover, the integration with other nanostructures, such as carbon nanotubes and palladium, is an excellent strategy to enhance the efficiency of electrocatalytic hydrogen evolution [59].

Despite the numerous and intriguing applications of titania NPs, issues about their release into the environment after their extensive use are being raised. Indeed, TiO_2_NPs toxicity is reported to affect not only microorganisms, algae and plants, but also animals because of the production of reactive oxygen species (ROS) and cell wall damages [60]. Decontaminations systems are, hence, necessary to limit the potential risks associated to the exposure of such nanomaterials. With this aim, supramolecular hydrogels have been designed to efficiently remove TiO_2_ nanoparticles with diameter below 50 nm from aqueous suspensions. The same system, moreover, was also able to trap quantum dots (QDs) and AuNPs [61]. Furthermore, the incorporation of TiO_2_NPs into a supramolecular hydrogel yielded a nano-hybrid gel with photo-switchable properties, conferred by titania NPs. The photocurrent generation of the system was enhanced with respect to the individual components, suggesting the potential use of such a material in optoelectronic devises with high conversion efficiency [62].

Even though they are out of the purpose of this review, some new cutting-edge applications of hydrogels for water desalination have to be mentioned. In this case, polymeric hydrogels serve as solar vapour generator, in which the solar energy is converted to evaporate water contained in the hollow pores of gel network. Hence, salinity of seawater sample is efficiently decreased under natural sunlight, without the aid of membranes or thermal distillation [63]. Moreover, gel phase conferred also antifouling properties to the solar adsorber. Photoactive materials typically used in polymeric gels for clean water generation include molybdenum carbide [64], titanium sesquioxide (Ti_2_O_3_) nanoparticles [65] and biomass-derived materials, such as konjac glucomannan and rice husk biochar [66,67].

### 3.2. Energy Conversion

On the basis of their network dimensions, gels can act both as support to optimise the efficiency of electrocatalysts and as active materials for electrocatalysis. In the former case, they are typically formed by micro- and meso- structures that provide porous networks to avoid the self-agglomeration of catalysts and to guarantee a more homogeneous dispersion of the active nanomaterials. Hence, they ensure a higher electrochemical surface area with overall better stability of the entire catalyst material [68]. For example, Stupp’s group reported that a supramolecular hydrogel, obtained by the self-assembly of a perylene derivative, provided a high surface-charge density and a porous substrate to stabilise a nickel-based catalyst and electrolytes for the photocatalytic production of hydrogen. They proved that the reaction did not occur in the absence of the gel phase, demonstrating the importance of the gel network to allow catalysts diffusion and consequential H_2_ evolution [69]. The presence of different hydrophilic functional groups of hydrogelators (such as carboxyl, hydroxyl or amino groups) increases the wettability of metal electrocatalysts and the oxidation of the metals in the active sites. For example, it has been recently reported that the incorporation of transition-metal oxides/hydroxides catalysts in N-doped carbon hydrogels increased wettability of the catalysts, facilitating the interactions between catalysts and aqueous electrolytes. As a result, a favourable oxygen evolution reaction (OER) activity was achieved, as demonstrated also by the high capacitive current density displayed by the gel catalyst [70].

**Figure 5:**
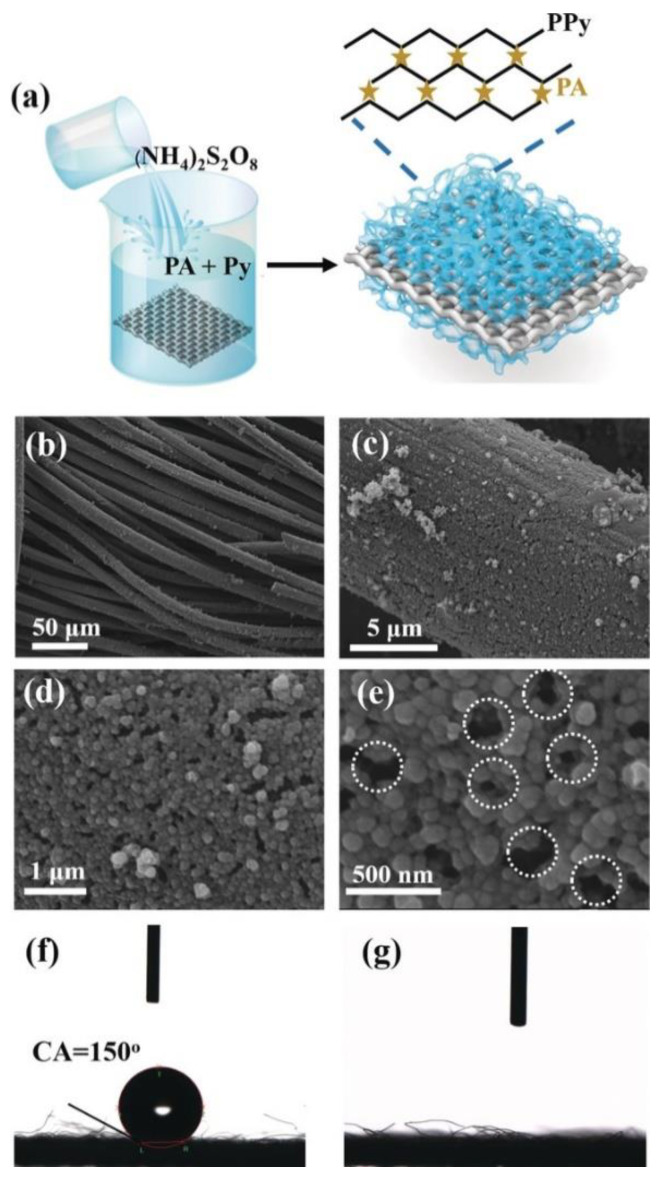
(**a**) Schematic representation of the production of phytic acid (PA)-doped polypyrrole (PPy) hydrogel coated on carbon cloth (denoted PA–PPy/CC); (**b**–**e**) SEM images of PA–PPy/CC with different magnifications; (**f**,**g**) contact angles of a drop of water on pure CC and PA–PPy/CC, respectively. Reprinted with permission form ref. [71] under the terms of the Creative Commons Attribution-Non-Commercial License. https://onlinelibrary.wiley.com/doi/full/10.1002/anie.201900109 [71].

On the other hand, when gels display nanosized networks, they can be employed as active and low-cost electrocatalysts in several reactions, such as OER, hydrogen evolution reaction (HER) and oxygen reduction reaction (ORR) [68]. This approach recently allowed the development of a conductive hydrogel with a superhydrophilic surface for metal-free and binder-free electrocatalysis. The superhydrophilicity favoured the contact between electrocatalysts and electrolyte, thereby improving the transport of electrolytes. In particular, the authors obtained a hydrogel from phytic acid and polypirrole that showed excellent OER activity with long term stability, fast charge and mass transport (Figure 5) [71]. Such a result could be particularly significant in the field of energy conversion, as the anode reaction OER is usually considered a big limitation in water splitting for the production of hydrogen and oxygen molecules in an electrolytic cell, because of the four-step proton-coupled electron transfer process that require high overpotentials (2H_2_O → 4H^+^ + O_2_ + 4e^−^ in acidic solution and 4OH^−^ → 2H_2_O + O_2_ + 4e^−^ in alkaline solution) [72]. In this way, they successfully boosted the OER with an innovative and low cost soft material, avoiding the use of metals, toxic chemicals and poorly sustainable processes like pyrolysis and calcination [71].

Alongside the OER, the HER occurs at the cathode of an electrolytic cell, generating gaseous hydrogen that is considered the ideal next-generation fuel, because of the zero-carbon footprint (water should be in theory the only product of H_2_ oxidation). Although HER occurs at a faster rate than OER, also in this case catalysts are required to overcome the activation energy barrier of the reaction (2H^+^ + 2e^−^ → 2H_2_ in acidic solution and 2H_2_O → 2OH^−^ + H_2_ + 2e^−^ in alkaline solution) that is a multi-step process based on the adsorption of hydrogen species, reduction and desorption of molecular hydrogen [68]. Platinum-based electrocatalysts are the benchmark catalysts, but they are being recently replaced by less expensive metals or even by metal-free materials, including carbon nanotubes and graphene [73]. For example, N-doped reduced graphene oxide (rGO) has been used to obtain hybrid hydrogels decorated with molybdenum disulfide (MoS_2_) nanosheets with efficient HER catalytic activity. MoS_2_ has been reported to have HER activity similar to Pt, but its tendency to aggregate reduces the exposed surface area and hinders its practical application as electrocatalyst. In this case, graphene hydrogel allowed a vertically aligned dispersion of nanosized MoS_2_, which displayed more active sites, and a high conductivity through the 3D hydrogel nano-architecture. This resulted in a high-performing HER catalyst with long-time catalytic stability [74].

## 4. Conclusions and Outlook

Creation of green energy conversion schemes is one of the biggest challenges of our society, strongly connected with planet future liveability and decent population life standards. In this perspective, gels can act as innovative emerging materials for energy and water technologies, offering their 3D networks as stable support for promoting ion and electron transfers in energy devices, such as electrolyte cells and energy harvesters. Both supramolecular and polymeric gels can enhance nanomaterial performances by limiting their aggregation propensity yielding composite multifunctional conductive materials with added properties, including flexibility and stretchability, which are two important features for the creation of ‘smart’ wearable devices with dynamic responsiveness. Hydrogel can also serve as modern processes of purification of water from toxic metal ions and nanoparticles, whereby a second and renewable use of such materials is exerted as heterogeneous catalysts in organo- and electro-catalysis. This approach is exceptionally compliant with recent international awakening in climate crisis, looking for green actions, sustainability and low impact behaviours. New energy technologies for the production of green fuels, such as H_2_ from solar energy or biomass [75], can provide excellent solutions and effective ways to lead urgent changes for environmental future health.
